# Reopening ECEC Services for Children Under Three Amidst the Pandemic: Investigating the Association of Health Measures with Pedagogical Practices and Children’s Well-being

**DOI:** 10.1007/s10643-023-01465-z

**Published:** 2023-04-05

**Authors:** Sara Barros Araújo, Rafaela Rosário, Ana Silva, Sílvia Barros

**Affiliations:** 1 Centre for Research and Innovation in Education - inED, ESE, Polytechnic of Porto, Rua Dr. Roberto Frias, 602, 4200-465 Porto, Portugal; 2 School of Nursing, University of Minho, UICISA:E Nursing School of Coimbra, Braga, Portugal; 3 Centre for Research and Innovation in Education - inED, ESE, Polytechnic of Porto, Porto, Portugal

**Keywords:** COVID-19 prevention and control measures, Centre-based services for children under three, Early childhood education and care, Pedagogical practices Children’s well-being

## Abstract

In Portugal, early childhood education and care services for children under-three were the first educational services to reopen after periods of lockdown. COVID-19 prevention and control measures had to be implemented nationwide, but no knowledge was yet produced on their impact in educational settings. This study aimed to map the implementation of COVID-19 prevention and control measures and examine associations among prevention and control measures, perceived changes to pedagogical practices and children’s well-being in early childhood education and care services for children under three. In this study, 1098 early childhood education and care professionals from all districts completed an online survey during January and February 2021. Results indicated that prevention and control measures were widely implemented. Furthermore, early childhood education and care professionals who started to implement prevention and control measures more frequently were more likely to perceive a reinforcement of their pedagogical practices at the level of adult-child interaction, emotional climate, and interaction with families, and reported higher levels of children’s well-being. Findings highlighted the potential role of pedagogical practices in mitigating the effects of COVID-19 in early childhood education and care services for children under-three.

## Introduction

After the declaration by the World Health Organization of a global pandemic caused by Severe Acute Respiratory Syndrome Coronavirus 2 (SARS-CoV-2), on the 11th March 2020 (WHO, 2020b), unprecedented changes in the lives of children and adults started to take place worldwide. International organizations and researchers have emphasized the detrimental effects of the COVID-19 pandemic for young children, including disruptions in daily routines across family life and early years educational settings (O’Keeffe & McNally, 2021); increased physical and psychological health vulnerability (Adıbelli & Sümen, 2020; Barnett & Jung, 2021; Egan et al., 2021; Shorer & Leibovich, 2022; Rundle et al., 2020; Ravens-Sieberer et al., 2021); increased poverty, deprivation, and inequality (Van Lancker & Parolin, 2020; UNICEF, 2020), mainly for those already facing disadvantaged or vulnerable situations (United Nations, 2020). Against this background, schools and educational services have been acknowledged as playing a critical role in supporting all children as countries attempt to tackle the repercussions of the pandemic on children, and to safeguard their rights to education, health and safety as set out in the Convention on the Rights of the Child (UNESCO et al., 2020).

In Portugal, ECEC services for children under three years of age were the first education services to reopen after periods of national lockdown (18th May 2020 and 15th March 2021, respectively). National debate arose on the critical challenges of balancing the measures for controlling the transmission of COVID-19 with the specificities of education and care practices with infants and toddlers. COVID-19 prevention and control measures (PCMs) issued by the Ministry of Health (Direção-Geral de Saúde [DGS], 2020) had to be implemented nationwide by ECEC services. Guidelines from the Ministry of Labour, Solidarity and Social Security (n.d.), which governs the 0–3 sector in Portugal, were also published prior to the services reopening. Both documents stressed containment measures and procedures to guarantee children’s and adults’ safety and health. No pedagogical guidelines were formally issued by the central government for the 0–3 sector on reopening services. Instead, pedagogical recommendations were published by a civil society network (Associação de Profissionais de Educação de Infância [APEI]), as the result of the collaborative work of early childhood teachers and academics, aiming to assure the pedagogical quality of childcare services when reopening these institutions (APEI, 2020).

Until now, few studies have focused on the reopening of ECEC services and the most part were centred on the impact of transmission and medical implications (e.g., Lachassinne et al., 2021; Haapanen et al., 2021). Despite the relevance of such a health-oriented approach, there is scant evidence about the associations of PCMs with the well-being and pedagogical experiences of infants, toddlers, and professionals working in this sector. Indeed, international guidelines on the reopening and operation of education services in pandemic circumstances have emphasized the need to actively monitor health indicators, focus on well-being and protection, and strengthen pedagogy (UNESCO et al., 2020).

The current study is part of a larger research project that addressed the educational and social impact of the COVID-19 pandemic in Portuguese ECEC services for children under three. Specifically, it aims to map the implementation of PCMs and explore associations among PCMs, pedagogical practices and children’s well-being, as reported by ECEC professionals. It intends to fill a gap in existing literature by contributing to generate knowledge on these unique and critical circumstances in order to inform future initiatives and assure the best conditions for early education and development.

## Implementation of COVID-19 PCMs in ECEC Centre-based Services

The reopening of ECEC centre-based services amidst the COVID-19 pandemic involved the implementation of COVID-19 PCMs worldwide. In Portugal, a study carried out in 16 preschools found that, in June 2020, a set of strategies were commonly implemented: to remove indoor and/or outdoor loose objects and materials that promoted gathering or sharing, to limit the number of children using the material at the same time, and to increase the use of outdoor space (Cordovil et al., 2021). Also, results indicated a decrease in implementing most health measures by September 2020, except for the use of outdoor spaces and disinfection measures.

A study with professionals working in 0–6 ECEC settings in Norway, Sweden, and the United States identified similar COVID-19 PCMs (e.g., extra handwashing, smaller group sizes, lower teacher-child ratios, stricter sanitation requirements, prohibiting parents from entering the centres, and social distancing) (Pramling Samuelsson et al., 2020). Participants from Norway and Sweden did not mention the use of masks. Professionals from the three countries reported that they felt ill-prepared for the logistics of implementing new hygiene and sanitation protocols. Furthermore, they expressed concern over some measures, such as social distancing, limited group size, separation of cohorts and, particularly, over children with special needs and those with a pre-identified vulnerable condition. However, teachers in Norway reported that smaller group sizes and more favourable adult-child ratios created the opportunity for them to interact with each child more often and follow-up on children’s interests more fully. Also, teachers in Norway and Sweden reported ethical struggles related to the desire to safeguard their health while fulfilling their professional responsibilities towards children and families. A duality in the emotional experiences of early childhood teachers’ working with children ranging from 0 to 5 years of age when returning to the classroom was also identified in the study by Sorrells and Akpovo (2022). Thus, ECEC teachers described feelings of joy and fear, the latter partially influenced by circumstances of the teachers’ return, namely the short time *to make the emotional and logistical transition to the new role as a “front-line” worker* (p. 11).

The study carried out by Atiles and colleagues (2021) on the reopening of preschools in the United States, in August 2020, reported the implementation of preventive measures such as masks, limitation to the number of visitors to the school, disinfection of surfaces and scheduling more hand washing. In the same study, participants from Latin American countries (Brazil, Costa Rica, Dominican Republic, Mexico, Paraguay and Puerto Rico) reported receiving training to return to face-to-face activities, including the establishment and application of protocols in the classroom, around school, and when working with families. Health personnel and nurses working in the institutions often supported these training initiatives. However, 48% of the participants indicated that the institutions where they worked did not provide training for the return to face-to-face care and education. In the study conducted by O’Keeffe and McNally (2021) with 310 professionals with past or present experience working in ECEC settings in Ireland, 42% reported concerns around the role of play as a pedagogical strategy upon children’s return given COVID-19 regulations. Furthermore, 23% expressed specific concerns on implementing and reinforcing safety protocols, such as social distance, sharing of toys, and hygiene procedures. Overall, professionals reported a sense of uncertainty about how to facilitate play in such circumstances, combined with concerns over the possibility of reverting to traditional methods. A sense of uncertainty and practical concerns with coping with social distance and possible lack of resources upon the reopening of schools were also reported by teachers working with older children in primary and secondary education (Kim et al., 2021). These teachers also revealed concerns over pupils’ well-being and academic progress. Overall, teachers’ identities mirrored centrally teachers’ caring natures, pupils-oriented priorities and *the sense of authentic interaction with pupils being at the heart of the profession* (p. 10).

## The Well-being of Children Under Three in Portuguese ECEC Settings

The education and care of children under three has been receiving growing attention in national and transnational agendas, mirroring a visible social and educational demand. High-quality centre-based services have been associated with short- and long-term positive effects on child development (e.g., Bratsch-Hines et al., 2020; Broekhuizen et al., 2018; National Institute of Child Health and Human Development [NICHD] Early Child Care Research Network, 2006; Pinto et al., 2019) and child well-being (Kalliala, 2011; OECD, 2018).

Research on infants and toddlers’ well-being in Portuguese centre-based settings is scarce, following an international tendency of lack of research on young children’s well-being in ECEC services (Mayr & Ulich, 2009; OECD, 2018). The majority of the studies carried out in Portugal have drawn on the experiential approach proposed by Laevers and colleagues (Laevers, 2000; Laevers et al., 2013) that defines well-being as a state that mirrors how the child’s basic needs, such as physical needs, need for tenderness, affection, safety, clarity, social recognition, to feel competent and need for meaning, are fulfilled has a result of both the quality of the educational environment and the children’s action (Laevers et al., 2005; Laevers & Declercq, 2018). Thus, the levels of child’s well-being allow for monitoring children’s socio-emotional development, their mental health and, ultimately, how their rights are being met (Laevers & Declercq, 2018). Under this framework, studies conducted in Portugal showed that infants and toddlers tended to exhibit positive indicators of well-being, from moderate (Araújo, 2012) to high levels (Carvalho, 2017), following empirical evidence found in international studies (Casas & Frønes, 2020; Laevers & Declerk, 2018). Evidence also suggested that well-being levels tended to be higher in toddler classrooms adopting a participatory pedagogic approach, in which the child’s autonomy, agency and decisions were centrally acknowledged, in comparison to classrooms with a transmissive-oriented approach, more centred on the adult’s agenda (Araújo, 2012; Machado & Oliveira-Formosinho, 2016). Moreover, the study carried out by Carvalho (2017) revealed that results reported by early childhood teachers tended to be more positive than results reported by external evaluators. Also, some evidence showed that the levels of children’s well-being tended to improve as a consequence of context-based in-service initiatives targeting the professional development of ECEC teachers (Araújo, 2012; Carvalho, 2017).

Finally, the study carried out by Peixoto and colleagues (2017) on the relationship between the implementation of transition practices from home to ECEC settings and the emotional well-being of infants found that the number of transition practices reported by ECEC teachers was positively associated with the emotional well-being of infants during the first month of attendance.

In spite of such promising empirical evidences, it’s important to note that the research carried out in Portugal on the well-being of children under three draws mainly on small-scale studies, with a limited number of participants. Otherwise, studies on the impact of critical life circumstances on infants’ and toddlers’ well-being, such as the ones lived throughout the pandemic, were not identified.

The present study finds its theoretical underpinnings on the experiential approach (Laevers, 2000; Laevers et al., 2013; Laevers & Declercq, 2018), emphasizing the core premise that the quality of the educational environment affects children’s experiences, including their state of well-being. Two research questions are examined: 1) What was the extent of the implementation of PCMs in ECEC settings for children under-three, as reported by ECEC teachers? 2) Is the implementation of PCMs associated with pedagogical practices and children’ s well-being, as reported by ECEC teachers? We hypothesize that: (1) The PCMs were widely implemented in ECEC settings for children under three, and (2) There are significant associations between the implementation of PCMs and pedagogical practices and children’s well-being.

## Method

### Participants

The current study is based on survey responses from 1098 early childhood professionals working in centre-based services for children aged 0 to 3 years old.

ECEC in Portugal is organized in a split system: one for children up to 3 years of age (i.e., infants and toddlers) and one for children aged between 3 and 6 years old, the age of enrolling in mandatory schooling. Services for younger children are regulated by the Ministry of Labour, Solidarity and Social Security, and services for 3–6, which are part of the national education system, are regulated by the Ministry of Education. Most services for infants and toddlers are provided by private institutions (for-profit and non-profit) (Gabinete de Estratégia e Planeamento/Ministério da Solidariedade, Emprego e Segurança Social [GEP/MSESS], 2019). The exception is the Autonomous Region of Madeira, where some public schools, coordinated by the Regional Secretariat of Education, Science and Technology, also include centre-based services for infants and toddlers. The most recent official report about services regulated by the Ministry of Labour, Solidarity and Social Security, reporting to 2018 (GEP/MSESS, 2019) and only about the mainland services, informed that: (a) ECEC services for 0–3 year-olds covered 48.4% of children; (b) centre-based care was substantially more used than formal family-based services; and (c) centre-based services were provided mainly by non-profit institutions, with only 24% of the centres belonging to the private for-profit sector.

ECEC professionals who participated in the current study were, on average, 38.9 (*SD* = 8.8) years old. Table 1 includes more information about professionals and about the centres they worked in, such as location and type of institution.

**Table 1:** Characterization of participants

	*n*	%
*Gender*		
Female	1091	99.5
Male	6	0.5
*Professional category*		
ECEC teacher	853	77.7
Assistant	245	22.3
*Education level*		
Secondary school level or less	213	19.4
Bachelor´s degree	591	53.8
Master/Doctorate’s degree	294	26.8
*Country Region*		
North	394	35.9
Centre	193	17.6
Lisbon and Tagus Valley	369	33.6
Alentejo	38	3.5
Algarve	47	4.3
Madeira	29	2.6
Azores	27	2.5
*Location of centre*		
Urban	674	61.4
Suburban	206	18.8
Rural	218	19.8
*Type of institution*		
Private non-profit	852	78.1
Private for-profit	239	21.9
*Children’s age group*		
Less than 12 months	124	11.4
12–24 months	415	38.2
24–36 months	420	38.6
Mixed group	128	11.8

### Measures

Data was collected through an online survey developed under the study. Preliminary versions of the questionnaire were piloted for comprehensibility, relevance, acceptability and feasibility throughout two phases, involving the feedback of experts and professionals from the target population and resulting in adjustments to the instrument.

For the purposes of this article, the following sections were used: (a) introductory section; (b) sociodemographic characterization and structural features of classrooms and childcare centres; (c) COVID-19 prevention and control measures (PCMs); (d) changes to pedagogical practices; (e) children’s well-being; and (f) overall appraisal.

The introductory section presented the general context of the study, including main objectives, institutional information (e.g., research centres involved), ethical procedures, expected time for completion of the survey, and declaration of voluntary and informed consent.

The second section included 19 questions about sociodemographic characteristics (e.g., age, years of professional experience), structural features of classrooms and childcare centres (e.g., number of children in the group and in the centre).

The three following sections, COVID-19 PCMs, changes to pedagogical practices, and children’s well-being, were developed under the study, following standardized procedures (de Vet et al., 2011; UNESCO, 2015) and considering existing literature and previous instruments related to the topics. The questionnaire about COVID-19 PCMs included 22 items written to capture the Portuguese Ministry of Health norms for childcare centres (DGS, 2020), allowing to score how frequently the measures were implemented. A 7-point scale, with response options ranging from “never” to “always”, was used. Principal Components Analysis (PCA) was used to assess the construct validity of implementing the PCMs about COVID-19, pedagogical practices and children’s well-being. Cronbach alpha served as a measure of internal consistency, reflecting the item’s average correlation within the scale (Cronbach, 1951). Through PCA, we found three dimensions (16 items total): hygienization and ventilation (α = 0.77); measures to assure physical distance (α = 0.72); and measures related to structural features (α = 0.64).

Items concerning changes to pedagogical practices were derived from pedagogical approaches for 0–3 education and care services (Oliveira-Formosinho & Araújo, 2018; Post, Hohmann & Epstein, 2011). Also, it included program quality evaluation instruments (Bertram et al., 2013; Hamre et al., 2014; Harms et al., 2006; HighScope Educational Research Foundation, 2013). Participants scored the changes in pedagogical practices during the pandemic time in the school year of 2020-2021 compared with the time before the pandemic. The 24 items were scored on a 7-item scale, ranging from “much less than before” to “much more frequent than before”. Four dimensions were found through PCA (24 items total): adult-child interactions (α = 0.92); emotional climate (α = 0.76); interaction with families (α = 0.78); and organization of space and materials (α = 0.46).

The questionnaire about children’s well-being included 17 items, partially based on Laevers and colleagues’ proposal (2005) and the Daycare Experience Questionnaire (Skouteris & Dissanayake, 2001). Participants were asked to indicate the proportion of children from their group that felt or displayed to feel according to each statement/item. Each item was scored on a 6-item scale, ranging from “all [children]” to “none”. We used one dimension (15 items total) with good internal consistency (α = 0.80). The survey also included two questions on overall appraisal. Professionals were asked to what extent did they agreed, or not agree, that the pandemic circumstances contributed to a decrease: (a) on the quality of pedagogical practices in childcare; and (b) on children’s well-being. A 7-point scale was used, with response options ranging from “totally disagree” to “totally agree”.

### Data Collection

Portuguese childcare centres from the mainland (all of the 18 districts), and autonomous regions of Azores and Madeira, with electronic contacts available online on the Social Chart (http://www.cartasocial.pt), in a total of 2 908 institutions, were contacted by e-mail, inviting ECEC professionals to participate in the online survey using the platform SurveyMonkey. In addition, the invitation to participate was reinforced through social networks and the APEI mailing list. Data was collected through the online survey from 23rd January to 21st February 2021. Participants completed the informed written consent form before completing the questionnaire. The Ethics Commission of the Centre for Research and Innovation in Education - inED approved the study (code PA8/CE/20).

### Data Analysis

Data analyses were performed using SPSS, version 28.0 (IBM, SPSS Inc. Chicago, IL), considering a level of significance of < 0.05.

Descriptive statistics were used to explore item-specific normality, and are presented as means, standard deviations (SD) and percentage (%). For the variables Implementation of the PCMs about COVID-19, Changes in pedagogical practices and Children’s well-being, two subgroups were created using the median split (below median versus above median).

Associations between the PCMs concerning COVID-19 (predictor variable) and changes in pedagogical practices, and children’s well-being (outcomes) were analyzed using logistic regression. Adjusted odds ratio (OR) with 95% confidence interval (CI) was considered to determine the strength of association between predictor and outcomes. As potential confounders, we included any variables hypothesized as affecting pedagogical practices, such as the ECEC professionals’ age, education level, years of experience, childcare centre localization, number of children in the room and in the childcare centre and, predominantly, children’s age.

## Results

### Implementation of COVID-19 Prevention and Control Measures

PCMs were widely implemented across the country (Table 2). The majority of the professionals reported that PCMs were always or often implemented. The most frequently implemented measures, said as always in place, were the use of personal protective equipment, the use of toys/materials provided exclusively by the centres, and the restriction of parents’ entry into the centres. Additionally, the results suggest that there was a concern with measures related to disinfection, with more than 90% of the participants reporting that these were always or often implemented: disinfection of surfaces (97.6%), disinfection of children’s hands (96.6%), and disinfection of materials (93.2%). Professionals also highly reported measures concerning ventilation and social distancing (namely the adoption of fixed groups of children and adults - “bubbles” - and compliance with circuits). Finally, a marked reduction of materials/toys usually accessible to children was reported in 81% of cases (always or often).

Despite the limitation of activities outside the centre (85.3% always or often), 69% of professionals reported that outdoor spaces were always often used. Results also indicate that the implementation of measures for children not to share toys/objects varied across settings, ranging from never (16.3%) to always (14.7%). Notably, the least reported measure was reducing the number of children per room, never implemented in 55.7% of cases.

Finally, although 97.1% of professionals acknowledged that information on PCMs was always or often available, 34.6% reported that training opportunities for professionals were never or rarely available (please see Table 2).

**Table 2:** Percentage of ECEC professionals reporting COVID-19 PCMs

	Always	Often	Sometimes	Rarely	Never
Use of personal protective equipment	96.4	3.2	0	0.2	0.2
Use of materials/toys exclusively of centre	83.5	13.4	1.4	1	0.7
Parents don’t enter centre	81.1	11.2	1	2.4	4.3
Information on measures is available	80.3	16.8	1.7	0.8	0.4
Assigned seats during meals	69.9	17.7	2.9	2.9	6.6
Limitation of activities outside centre	68.5	16.8	3.4	3.8	7.6
Disinfection of surfaces (contact points)	69.8	27.8	1.8	0.6	0.1
Disinfection of materials	60.5	32.7	4.3	1.8	0.8
Disinfection of children’s hands	59.6	37	2.6	0.4	0.5
Ventilation of rooms, hallways	57.2	37	4.3	0.9	0.6
Separate, fixed groups of children (“bubbles”)	50.5	34	5.3	4.2	6.1
Compliance with circuits	45.8	34.3	7.4	6.3	6.2
Physical distancing between children (tables, cradles)	39.1	36.2	9.3	8.8	6.6
Marked reduction in materials/toys	38.3	42.8	10	4.8	4.2
Staggered schedules	35	33.1	8.7	9.6	13.8
Use of outdoor spaces	28.6	40.4	14.6	9.9	6.6
Activities in small groups	23.9	47.2	16.4	7	5.8
Training opportunities for professionals	18.9	28	18.6	19	15.6
Measures for children not to share toys/objects	14.7	35.6	15	18.4	16.3
Reduction in number of children per room	8	14	10	12.3	55.7

### Associations Between PCMs and Changes in Pedagogical Practices

Professionals who implemented PCMs more frequently reported that they had reinforced their pedagogical practices in adult-child interaction and interaction with families, even after adjusting for professionals’ age, professional category, educational level, professionals’ years of experience, childcare centre location, number of children in the room, number of children in the centre and children’s age (please see Table 3).

**Table 3:** The associations between PCMs and reinforcement of pedagogical practices

	Disinfection and ventilation OR (95%; CI)	Social distancing OR (95%; CI)	Centre/classroom organisation OR (95%; CI)
Adult-child interaction	**2.7 (2.0; 3.5)**	**2.0 (1.5; 2.6)**	**2.0 (1.6; 2.6)**
Emotional climate	**1.9 (1.5; 2.6)**	1.2 (0.9; 1.5)	**1.4 (1.1; 1.8)**
Interaction with families	**2.3 (1.8; 3.1)**	**2.2 (1.7; 2.9)**	**2.2 (1.7; 2.9)**

Adjustment variables: professionals’ age, professional category, education level, years of experience, childcare centre localization, number of children in the room and in the childcare centre, and children’s age

p values < 0.05 are given in bold

The results suggest an effort to reinforce pedagogical practices; however, 57% of professionals agreed that the pandemic might have contributed to reducing their quality when asked for an overall appraisal of the pandemic’s impact on pedagogical practices.

### Associations Among PCMs, Pedagogical Practices and Children's Well-being

ECEC professionals perceived positive signs of well-being in children. According to their perceptions, all or almost all children showed signs of physical or emotional well-being (e.g., satisfaction/joy during activities, in interacting with other children, good mood throughout most of the day), except for appetite changes.

Despite these results on the well-being of infants and toddlers attending their classroom, when asked for an overall appraisal of the pandemic’s impact on children’s well-being in childcare, 29.3% of professionals considered that the pandemic might have contributed to a decrease in children’s well-being.

Associations between PCMs and children’s well-being indicated that, after adjustment for confounders, the professionals who frequently implemented the disinfection and ventilation measures were more likely to perceive positive indicators of children’s well-being. However, there were no significant associations between the reported implementation of PCMs related to social distancing and centre/classroom organisation and perceived children’s well-being.

Regarding pedagogical practices during the pandemic, professionals who reported to have increased the quality of adult-child interaction and the emotional climate in the classroom were more likely to report positive indicators of children’s well-being, even after adjusting for confounders (please see Table 4).

**Table 4:** Children´s well-being, PCMs and pedagogical practices

PCMs	Disinfection and ventilation OR (95%; CI)	Social distancing OR (95%, CI)	Centre/classroom organisation OR (95%, CI)
Children´s well-being	**1.8 (1.4; 2.4)**	1.3 (0.9; 1.7)	1.2 (0.9; 1.5)

Pedagogical practices	Adult-child interaction OR (95%; CI)	Emotional climate OR (95%; CI)	Interaction with families OR (95%; CI)
Children’ s well-being	**1.7 (1.3; 2.2)**	**1.7 (1.3; 2.2)**	1.3 (0.9; 1.7)

Adjustment variables: professionals’ age, professional category, education level, years of experience, childcare centre localization, number of children in the room and in the childcare centre, and children’s age

p values < 0.05 are given in bold

## Discussion

This study aimed to map the implementation of COVID-19-related PCMs in centre-based services for infants and toddlers in the Portuguese context, as reported by professionals (ECEC teachers and assistants) working in these settings. Additionally, associations were explored between the implementation of these measures, pedagogical practices and children’s well-being.

We found that the vast majority of professionals implemented COVID-19 PCMs in their centres/classrooms, confirming our first hypothesis. These results follow a tendency previously identified in 0–6 settings in Norway, Sweden and the United States (Pramling Samuelsson et al., 2020) and in preschools in the United States (Atiles et al., 2021) and Portugal (Cordovil et al., 2021). However, variations across studies were identified on specific PCMs. Thus, the most frequently reported PCM in our research, the use of personal protective equipment, was also reported by teachers in the United States (Atiles et al., 2021) but not by teachers in Norway and Sweden (Pramling Samuelsson et al., 2020). Meanwhile, the reduction in the number of children per room was the least reported PCM in our study, while it was reported as one of the adopted measures by teachers working in 0–6 settings in Norway, Sweden and the United States (Pramling Samuelsson et al., 2020).

Restrictions to parents entering the centres, a PCM primarily reported by the participants in our study, was also reportedly implemented in Norway, Sweden, and the United States (Pramling Samuelsson et al., 2020). Disinfection measures were also put in place in these three countries, which is in line with the results of our study. Furthermore, a study conducted in Portuguese preschool settings indicated that disinfection measures increased between June and September 2020 (Cordovil et al., 2021). Moreover, results suggested that disinfection measures had a higher positive perception among preschool teachers because they did not hinder children’s opportunities for play, such as the limitation on using and sharing materials among children. Notably, the measures for children not to share toys/objects were one of the least reported by professionals in our study, in contrast with a very significant report of disinfection practices in childcare centres/classrooms. This may indicate that the implementation of PCMs was also conveying pedagogical criteria, and not a strict compliance to sanitary norms that could compromise children’s well-being, learning and development.

Finally, even if 97% of the professionals acknowledged the availability of information on PCMs in their centres, more than one third reported that training opportunities on COVID-19 and associated topics were never or rarely available, regardless of the guidelines of the Direção-Geral de Saúde (2020) that stressed the need to train all professionals before the reopening of services. The lack of training to return to face-to-face activities was also reported by 48% of professionals working in early education programs in the study carried out by Atiles and colleagues (2021) in the United States and Latin American countries. These pieces of evidence may demand careful reflection, once the professionals were faced with unprecedented changes, reporting uncertainty about the reopening and operation of educational services, concerns about children’s well-being and learning (Kim et al., 2021), ethical struggles on how to safeguard their own health while taking on their professional responsibilities (Pramling Samuelsson et al., 2020), and duality in emotional experiences characterized by joy and fear (Sorrells & Akpovo, 2022).

Findings also suggest that professionals implementing PCMs more frequently (except social distancing) were more likely to report a reinforcement of their pedagogical practices concerning adult-child interaction, emotional climate in the classroom and interaction with families. Those professionals who used more social distancing measures were not significantly associated with a reinforcement of their pedagogical practices. It is possible that those professionals who implemented more social distance measures were more likely to represent emotional climate change more distant and accepting it (McDonald et al., 2015). Also, it is possible that they socially ward off the COVID-19 threats (Maiella et al., 2020).

We know that professionals’ concerns with the possible negative consequences of the implementation of COVID-19 PCMs (namely, social distancing, sharing of toys and hygiene procedures) in ECEC contexts were expressed in prior research (O’ Keeffe & McNally, 2021). Our findings indicate that professionals with the higher implementation of PCMs perceived themselves as having reinforced their pedagogical practices, possibly as a protective strategy throughout multiple and demanding changes. In Portugal, centre-based services for children ages 0–3 were operating in their maximum capacity from 18 May 2020 to 21 January 2021, a period in which professionals may have built capacity in balancing the implementation of PCMs while safeguarding the quality of adult-child interactions and emotional climate. Prior results seem to support this evidence of a growing professional competence to accommodate health demands while trying to ensure pedagogical quality (Cordovil et al., 2021).

Only the implementation of disinfection and ventilation was significantly associated with children´s well-being. As argued before, it seems that those professionals implementing these measures more often were particularly concerned with providing a less restrictive pedagogical setting, by increasing the opportunities for children’s to engage with toys and other pedagogical materials, and to be involved in social exchanges with other children. In the light of the experiential approach (Laevers, 2000; Laevers et al., 2013; Laevers & Declercq, 2018), these less restrictive circumstances could have subsidized the quality of children’s educational experiences and, by that, contributed for their state of positive well-being.

Despite these results, when asked for an overall appraisal, more than half of the participants in our study agreed that the COVID-19 circumstances may have contributed to a decrease in the quality of pedagogical practices in centre-based services for infants and toddlers. Indeed, the extensive implementation of some PCMs in Portuguese settings, namely the use of personal protective equipment, restrictions to parents access to centres, fixed group (“bubbles”) or the marked reduction of toys/objects, may have hindered interaction and communication between children and adults, among children and among adults, in a critical microsystem for children’s development and learning (e.g., Burchinal, 1999; Campbell et al., 2008; NICDH ECCRN, 2001, 2006). However, the extent of this impact is yet to be determined.

Notably, professionals reported high proportions of infants and toddlers presenting physical and emotional well-being indicators in their classrooms, confirming previous results in the Portuguese context (Araújo, 2012; Carvalho, 2017). These findings are very relevant, taking into consideration that the COVID-19 pandemic caused increased physical and psychological health vulnerability in children (Adıbelli & Sümen, 2020; Barnett & Jung, 2021; Egan et al., 2021; Shorer & Leibovich, 2022; Rundle et al., 2020; Ravens-Sieberer et al., 2021). Also, it is paramount to ensure the current and future mental health and well-being of young children affected by the pandemic (Bertram & Pascal, 2021). Indeed, concerns over prioritizing children’s well-being during the reopening of educational services were expressed by ECEC professionals (Kim et al., 2021; Pramling Samuelsson et al., 2020), given the unprecedented changes that had to take place. Our findings suggest that the professionals having reported a reinforcement of their practices at the level of adult-child interaction and emotional climate were more likely to report positive indicators of children’s well-being, which may point to the mitigating effect of those variables in children’s well-being. Indeed, some literature acknowledges that the quality of adult-child interaction plays an important role on children under-three’s well-being (e.g., Kalliala, 2011). Our findings suggest that both, adult-child interactions and emotional climate, may have been critical in meeting the basic needs of infants and toddlers, in protecting their well-being and their rights (Laevers & Declercq, 2018). These pieces of evidence add to previous findings on the role of educational settings in guaranteeing children’s well-being in times of crisis (O’ Keeffe & McNally, 2021) and contribute to answer to recommendations of the WHO (2020a) on the need to carefully monitor the impact of school reopening, specifically the effects of policies and measures on the health and well-being of children, not only when they return, but also as they progress through school (Sonnenschein & Stites, 2021; Stites et al., 2021).

## Limitations of the Study and Conclusions

To the best of our knowledge, this is among the first studies to focus on the impact of the COVID-19 pandemic in ECEC services, thus adding uniquely to the previous research about education and well-being in the early years. However, some limitations should be recognised when interpreting results. First, it is essential to acknowledge that this cross-sectional study was based on professionals’ reports about their pedagogical and health practices, and children’s well-being in their classrooms. Consequently, the possible effect of social desirability should not be dismissed, as there is the possibility of increased scores due to perceived social pressure. Nevertheless, we should note that (a) professionals’ participation was voluntary; (b) introduction to the survey clarified that all answers would be confidential; and (c) questionnaires were responded to anonymously and were self-completed, in moments and places chosen by participants. Those conditions may offer some protection from this potential limitation. Second, given the cross-sectional nature of the study, cause and effect relations should not be drawn from findings. Third, though this study used a convenience sample, the high number of respondents, distributed across all the districts of Portugal, contributes to the results’ trustworthiness. Finally, further research may include other informants, as parents, other methods for data collection, such as observation in ECEC settings, and a combination of self-reported and external observer measures.

Despite these limitations, this study expands scientific knowledge on the pandemic and its effects, by focusing on an age group (i.e., 0–3 years old) understudied in research about the COVID-19. Furthermore, it brings an interesting approach by focusing on pedagogical practices during the pandemic, and not only on health topics. Findings suggest that PCMs were widely implemented in centre-based ECEC services for children below 3 in Portugal. Higher reports on the implementation of PCMs were positively associated with the perceived reinforcement of pedagogical practices. Furthermore, ECEC professionals who were perceived to have reinforced their practices, namely in adult-child interaction and emotional climate, reported to observe higher levels of child well-being in their classrooms. Considering the importance of high-quality practices in ECEC for child development and well-being, initiatives aiming at fading the effects of the COVID-19 pandemic should consider the potential protective effect of pedagogical practices. Particularly, given the nexus between the overall quality of pedagogical practices and the preparation of the ECEC workforce (European Commission, 2014; OECD, 2018), pre-service and in-service professional education and training initiatives should be given high priority. In Portugal, the public policies for the 0–3 sector have been characterized by an investment on the access to these services, but studies have also been acknowledging the need for a public investment in the pedagogical qualification of the sector (Araújo, 2012, 2017; Barros & Aguiar, 2010; Pinto et al., 2019). The results of our study show that the importance of such qualification is maximized in times of crisis, characterized by an intensification of professional demands and by potential threats to children’s well-being and learning. Indeed, it is important to note that more than 1/4 of professionals considered that the pandemic might have contributed to a decrease in children’s well-being in childcare. Concerns about mental health have been raised during this particular historical moment, and measures to improve living and education conditions should be prioritized right from the early years and covering all their life contexts. ECEC professionals are crucial allies to foster a better society, as they interact with children in a particularly important period for their education and development. Positive signs may be drawn from this data, as these professionals appeared to be confident that measures to limit the pandemic and to protect children’s well-being by reinforcing the quality of education and care, were put in place.
